# Supplementary material for: Splice-Junction-Based Mapping of Alternative Isoforms in the Human Proteome
Source: Cell Rep. Author manuscript; Available in PMC 2020 Jan 15. (PMC6961840; doi:10.1016/j.celrep.2019.11.026)
Supplement: 2 [file NIHMS1546469-supplement-2.zip › DF1/PXD006675/SmoothMuscleCells/SmoothMuscleCells_15_NASP_AAVEGTVEAGATVESTALK.pdf]

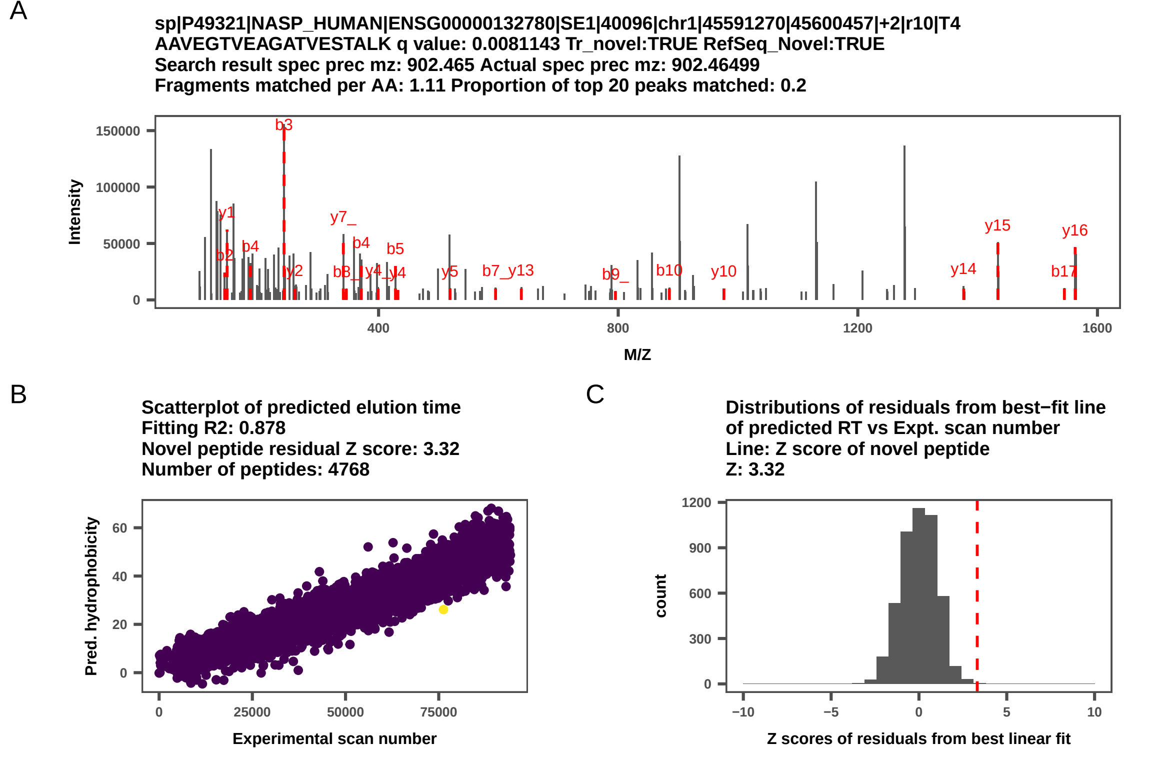
A
sp|P49321|NASP_HUMAN|ENSG00000132780|SE1|40096|chr1|45591270|45600457|+2|r10|T4
AAVEGTVEAGATVESTALK q value: 0.0081143 Tr_novel:TRUE RefSeq_Novel:TRUE
Search result spec prec mz: 902.465 Actual spec prec mz: 902.46499
Fragments matched per AA: 1.11 Proportion of top 20 peaks matched: 0.2
Intensity
M/Z
b3
y1
b2
b4
y7_
b4
b5
y2
b8
y4
y4
y5
b7_y13
b9_
b10
y10
y15
y16
y14
b17
B
Scatterplot of predicted elution time
Fitting R2: 0.878
Novel peptide residual Z score: 3.32
Number of peptides: 4768
Pred. hydrophobicity
Experimental scan number
C
Distributions of residuals from best−fit line
of predicted RT vs Expt. scan number
Line: Z score of novel peptide
Z: 3.32
count
Z scores of residuals from best linear fit
